# Student and teacher perceptions of community of inquiry in hybrid virtual classrooms

**DOI:** 10.1016/j.heliyon.2022.e12549

**Published:** 2022-12-24

**Authors:** Tjark Huizinga, Anne Lohuis, Judith Zwerver-Bergman, Rosalien van der Meer

**Affiliations:** a Saxion University of Applied Sciences, School of Education, Department of Innovative and Effective Education, P.O. Box 70.000, 7500KB, Enschede, the Netherlands; b Saxion University of Applied Sciences, Saxion Parttime School, Department of Quality Management and Research, P.O. Box 70.000, 7500KB, Enschede, the Netherlands; c Saxion University of Applied Sciences, Education and Student Support, Teaching and Learning Centre / School of Health, P.O. Box 70.000, 7500KB, Enschede, the Netherlands

**Keywords:** Distance education and online learning, Teaching/learning strategies, Learning spaces, Educational presences

## Abstract

The COVID-19 period forced (higher) educational institutions to come up with new ways to offer courses. This study focuses on hybrid virtual classrooms, which are learning environments that provide learning activities, guided by a teacher, to both *online* and *onsite* students simultaneously. Part-time students evaluated, based on their experiences, how hybrid virtual classrooms stimulated their feeling of being part of a community of inquiry [CoI]. They related this to the three aspects of CoI: social, teaching, and cognitive presence. Additionally, teachers' perceptions on how they enhanced the three aspects were collected. A mixed-method approach was applied in which a validated self-report questionnaire to measure the experienced CoI feeling of students was used. Qualitative data were collected through interviewing teachers about their experiences. The results illustrate that students value hybrid virtual classrooms. Students expressed that social presence can be further enhanced, especially by improving communication tools. Teachers applied various strategies to enhance social presence but felt limited by the communication tools. This also affected their opportunities to stimulate student interaction. Students felt more motivated to engage in deeper learning and felt supported by teachers in their learning process. In conclusion, students and teachers both value hybrid virtual classrooms, but enhancing social presence is challenging. To overcome this, teachers require a better understanding of meaningful learning activities to stimulate interaction between online and onsite students.

## Highlights

• During (intelligent) lockdowns hybrid virtual classrooms can stimulate student connectedness.
• Maintaining and engaging both *online* and *onsite* remains challenging.
• Hybrid virtual classrooms require additional pedagogical strategies from teachers.
• Classroom set-up affects efficiency for teachers to fulfill their preferred teacher role.
• Communication and student engagement depend on the quality of the technology.

## 1 Introduction

The COVID-19 pandemic offered higher educational institutes [HEIs] various opportunities and challenges for facilitating student learning (e.g., Kalmar et al., 2022; Sunitha et al., 2022; Vital-López et al., 2022). One of the main challenges was improving the usage of online learning to overcome the limitations of group sizes in classrooms. Given the COVID-19 (national) restrictions and legislations, HEIs were looking for ways to continue teaching and align their education to students' needs. In the Netherlands, as in other countries worldwide (e.g., Raes, 2022; Triyason et al., 2020), group sizes were limited because of the social distancing legislation. For HEIs in the Netherlands, who offer courses to both full-time and part-time students, this affected the way courses were offered. This study focuses on teachers and students in the part-time education context of one large HEI in the Netherlands.

After the first lockdown (in March 2020), approximately 70 percent of the part-time students of this HEI articulated the need for more opportunities for face-to-face education. However, at the peak of the pandemic maximum group sizes were introduced. As a result, innovative solutions to continue teaching and learning were required. Educational designers and planners of the HEI developed various scenarios aligned to the national legislation. The most fruitful scenario was the introduction of hybrid virtual classrooms [HVC]. An HVC is a learning environment in which students can either join onsite or online during synchronous classroom meetings (Raes et al., 2020a). Within the constraints of social distancing and maximum group sizes, HVCs offered opportunities for students to engage in (virtual) face-to-face education. Furthermore, recent studies on synchronous HVCs express cautious optimism regarding the opportunities for a more flexible, engaging learning environment compared to fully online instruction. This raises the question how these environments can be shaped as effectively as possible. Such a new learning space has several challenges: both pedagogical and technological (Raes et al., 2020a).

By introducing HVCs, the HEI aimed to enhance students' social presence and to further engage learners in the learning process during the COVID-19 pandemic. One of the key characteristics of the educational philosophy for part-time education of the HEI is supported peer learning, since the philosophy is grounded in social constructivism and flipped classroom. While learning, students are engaged in learning communities of the course(s) they attend. Within these learning communities, students' social and cognitive presence and teaching presence play a vital role (Garrison et al., 2001; Castellanos-Reyes, 2020). The alignment of these presences has been defined in the Community of Inquiry [CoI] model (Garrison et al., 2001; Shea et al., 2022).

Although various studies applied the CoI-model within (massive) online (open) courses (e.g., Cohen and Holstein, 2018; Kaul et al., 2018; Thymniou and Tsitouridou, 2021), little is known about how learning communities in synchronous HVCs are experienced by part-time studies related to the three presences (Stenbom, 2018). Therefore, this study aims to identify how part-time students experience the CoI-feeling when engaged in synchronous HVCs and how teachers try to enhance this CoI-feeling.

## 2 Theoretical framework

The theoretical framework consists of the two main themes of this study, namely characteristics of Hybrid Virtual Classrooms and the Community of Inquiry-model.

### 2.1 Hybrid virtual classrooms: opportunities and threats for flexibility

HVCs consist of students who are physically present in the classroom (onsite) and students who attend learning activities simultaneously online. HVCs can, therefore, be seen as a form of blended learning (Osguthorpe and Graham, 2003). Often HVCs are introduced to facilitate learning for both online and onsite students by offering them courses synchronously (Raes, 2022; Raes et al., 2020b). As the study of Raes and colleagues (2020b) illustrated, HVCs offer benefits for teaching and learning from both an *organizational* and a *pedagogical* perspective. From an *organizational* perspective, the core element of HVCs is that access to education becomes less dependent on a specific place. This offers opportunities for a broader student population to attend lectures and interact with other students and the teacher. Additionally, introducing HVCs helps students to reduce travel time and provides opportunities for students who suffer from illness to attend lectures. Furthermore, teaching efficiency can be increased, since offering courses through HVCs prevents that courses are offered in various locations or that classroom capacity needs to be expanded (Raes et al., 2020a, 2020b). From a more *pedagogical* perspective, HVCs provide opportunities for richer learning experiences, since including external experts becomes easier, because they are less dependent on time and place. Furthermore, collaboration between a broader range of (fellow) students becomes much easier than in traditional face-to-face settings. Additionally, students can decide how to attend classes and align this to their learning preferences and practical boundaries, so they do not have to miss learning activities (Bower et al., 2015; Raes et al., 2020a, 2020b).

Apart from these opportunities, there are also serious challenges that must be dealt with when using HVCs. Both Bower et al. (2015) and Raes et al. (2020a, 2020b) identified pedagogical challenges from the teacher and student perspective as well as technological challenges. Synchronous hybrid education requires new teaching methods and skills in using technology. In addition, overseeing and coordinating an HVC is more complex than a classroom in a full onsite or online setting. Therefore, teachers need opportunities for professional development and experimentation in their classrooms (i.e. Desimone, 2009). From a student perspective, online students are at risk of feeling less engaged, less included, and experiencing difficulties in getting the teacher’s attention. The studies of Raes (2022) and Raes et al. (2020b) empirically endorse this by showing that aspects such as motivation, relatedness to peers, sense of presence and sense of belonging are challenges for the online group. The study of Meer et al. (2021) underlined the importance of social connectedness for learning. Moreover, active participation in an HVC demands more self-discipline from online students.

From a technological stance, video and audio quality are essential for the learning experience of particularly online students. At the same time, the awareness of being recorded can influence teachers' behavior. Lastly, problems, limitations, or changes in devices and other technologies that are used in the HVC can be frustrating and hinder the learning process (Raes et al., 2020a). These kinds of technical issues are also addressed by Triyason et al. (2020) who in their study show that technical inequality can obstruct the effectiveness of hybrid virtual classrooms. Raes (2022) stresses the importance of the physical set up of the tools and learning space as it can be related to student engagement by making interactions possible.

Since only few empirical studies address synchronous learning activities in flexible hybrid forms, additional research is needed to gain more insight into the impact on student learning and the effectiveness of various instructional interventions in this context (Raes et al., 2020a, 2020b). The experiences of both students and teachers should be further investigated (Raes, 2022).

### 2.2 Community of inquiry-model

Around the start of the millennium, the Community of Inquiry-model was introduced as a conceptual framework to help educators design meaningful online courses (Garrison et al., 2000, 2001). Garrison (2011, p. 2) defined a Community of Inquiry as “*a group of individuals who collaboratively engage in purposeful critical discourse and reflection to construct personal meaning and confirm mutual understanding*”*.* This can only be achieved if learners within a(n online) course are actively engaged in meaningful learning activities and feel socially connected. In the original model, three presences have been identified that help to stimulate the CoI-feeling. As Shea et al. (2022, p. 149) clarify “*the constructs are termed* “*presences*” *to reflect their distribution across the people and materials in interaction*”. The three presences are: 1. Social presence, 2. Teaching presence, and 3. Cognitive presence (Garrison et al., 2001; Stenbom, 2018). As the study of Stenbom illustrated, additional presences were introduced, including learner, emotional, and autonomy presences, but none are validated to fit the framework. Therefore, the original model still stands until today and is shaped as a Venn-diagram to also illustrate the overlap between presences and the consequences for educational design.

For an optimal educational experience, the presences need to be aligned (Garrison et al., 2001; Swan et al., 2008; Stenbom, 2018; Shea et al., 2022). Therefore, design decisions made for one presence affect the other presences. Additionally, students and teachers have a shared responsibility to develop a meaningful CoI (Shea et al., 2022) and need to determine how to support discourse, regulate learning and set a (safe) learning climate. Whereas the initial model was developed for asynchronous online learning, more recent studies also apply it in blended learning designs (e.g., Le Roux and Nagel, 2018; Hilliard and Stewart, 2019). This was also shown by Stenbom (2018) who illustrated that little research is conducted within the context of part-time students and the implementation of HVCs.

#### 2.2.1 Social presence

Social presence refers to the degree to which learners feel socially and emotionally connected with others in online learning environments (Swan et al., 2008; Castellanos-Reyes, 2020; Chen, 2022). Within full online environments this can be challenging since learners are not familiar with peer learners at the start. Therefore, including (in)formal activities helps to better understand the backgrounds and perspectives of learners. Also, in blended synchronous course designs social connectedness is a prerequisite for creating learning opportunities within communities (Meer et al., 2021). Additionally, the community feeling is enhanced when learners feel that they are a substantive part of the community itself. This requires that learners communicate with peers in the learning environment (Shea et al., 2022) and that the communication is meaningful (Chen, 2022). Furthermore, all participants involved in the (online) learning environment (teachers, learners and expert) are expected to engage in open communication and be trustworthy (Molano and Polo, 2015). By enhancing interaction with peers, the inter-personal relationship will grow (Wang, 2005). The inter-personal relationship is a prerequisite for learners to engage in peer-interaction and peer-teacher interactions.

Several studies showed that online students still felt excluded from the chief class (onsite students) because they were physically separated (Raes et al., 2020a). Interaction between participants is necessary for HVCs. But interactions themselves are not sufficient to ensure effective online learning. These types of interactions need to have clearly defined parameters and need to be focused on a specific direction, hence the need for teaching presence (Raes et al., 2020a).

#### 2.2.2 Teaching presence

Teaching presence reflects how teachers design, facilitate and support cognitive and social processes to achieve meaningful learning (Garrison et al., 2001). As Anderson et al. (2001), Garrison (2017) and Stenbom (2018) described teaching presence consists of three elements: instructional design and organization, facilitation of discourse, and direct instruction. Chen (2022) adds that teaching presence involves all activities that teachers include in a course to stimulate the learning process, such as providing feedback and encouraging students.

The instructional design and organization refers to setting up the curriculum, defining how long learners can learn (time limitation) and how to utilize the online medium in a meaningful way (Shea and Bidjerano, 2009; Shea et al., 2010). Additionally, for meaningful discourse, teaching presence also refers to how productive discourse is stimulated. From this stance, teachers identify (dis)agreements, seek consensus and understanding, develop a learning climate, and include participants in the learning process. The final aspect of teaching presence relates to direct instruction*,* which teachers enhance by presenting content, focusing on discussions, summarizing understanding, and responding to technical issues.

As part of the teaching presence, teachers need to develop ways of enhancing interaction between onsite and online learners. They need to find ways to promote learning for all students involved, to engage learners in meaningful interaction with each other and to promote deep understanding.

#### 2.2.3 Cognitive presence

Cognitive presence refers to “*the exploration, construction, resolution and confirmation of understanding through collaboration and reflection in a community of inquiry*” (Garrison, 2007, p. 65). Within a CoI, cognitive presence is required to gain new insights by, for instance, reflecting on actions and outcomes. Various studies demonstrated that students’ curiosity and interest in the course topic itself are a bandwagon for their cognitive presence (e.g., Garrison et al., 2001; Swan et al., 2008; Hilliard and Stewart, 2019). Moreover, the various stages of an inquiry cycle (including finding information, various perspectives, finding answers) are key components to stimulate the cognitive presence.

Within CoI, cognitive presence is enforced when students feel connected with each other. Therefore, social presence and cognitive presence combined results in discourse within the learning environment. By offering guidance, for example, through learning activities, the discourse can be enhanced. Therefore, teaching presence is required to develop meaningful discussions and discourse within CoI.

## 3 Context of the study

In this section, relevant contexts of the study are described. This concerns the characteristics of part-time education at the specific HEI where this study took place and the preparation of HVCs during the COVID-19 pandemic.

### 3.1 Flexible part-time education in the Netherlands

Within the specific large-scale HEI, the faculty for part-time education aims to offer programs that meet the needs of working professionals and job seekers. This calls for flexible education aligned with students' professional practice and helping them to prioritize efficient and flexible learning (e.g., Lohuis et al., 2021a).

The faculty aims at using a blended learning approach, in which face-to-face, online, and workplace learning are integrated. From a pedagogical stance, the flipped-classroom approach was chosen. This was contextualized by offering students opportunities for time and place-independent learning prior to classroom meetings. This was possible since all relevant learning activities are provided beforehand through the electric learning environment. During classroom meetings, learning activities are included to deepen students' understanding of the content and to collaboratively share and reflect on experiences with fellow students.

### 3.2 Preparing virtual hybrid classrooms during COVID-19 pandemic

In the HEI a pilot with HVCs for students in the field of Economics and (Social) Health Care was initialized. A team from the part-time faculty, including one of the authors of this study, worked together with multimedia (learning) experts to prepare classrooms and teachers. From September 2020 the pilot started and students could alternately attend classes online and onsite.

For enhancing the implementation process of HVCs, it was identified what support was needed in the following three areas: 1) technical support, 2) pedagogical support and 3) organizational support (Lohuis et al., 2021b).

#### 3.2.1 Technical support

**Figure 1:**
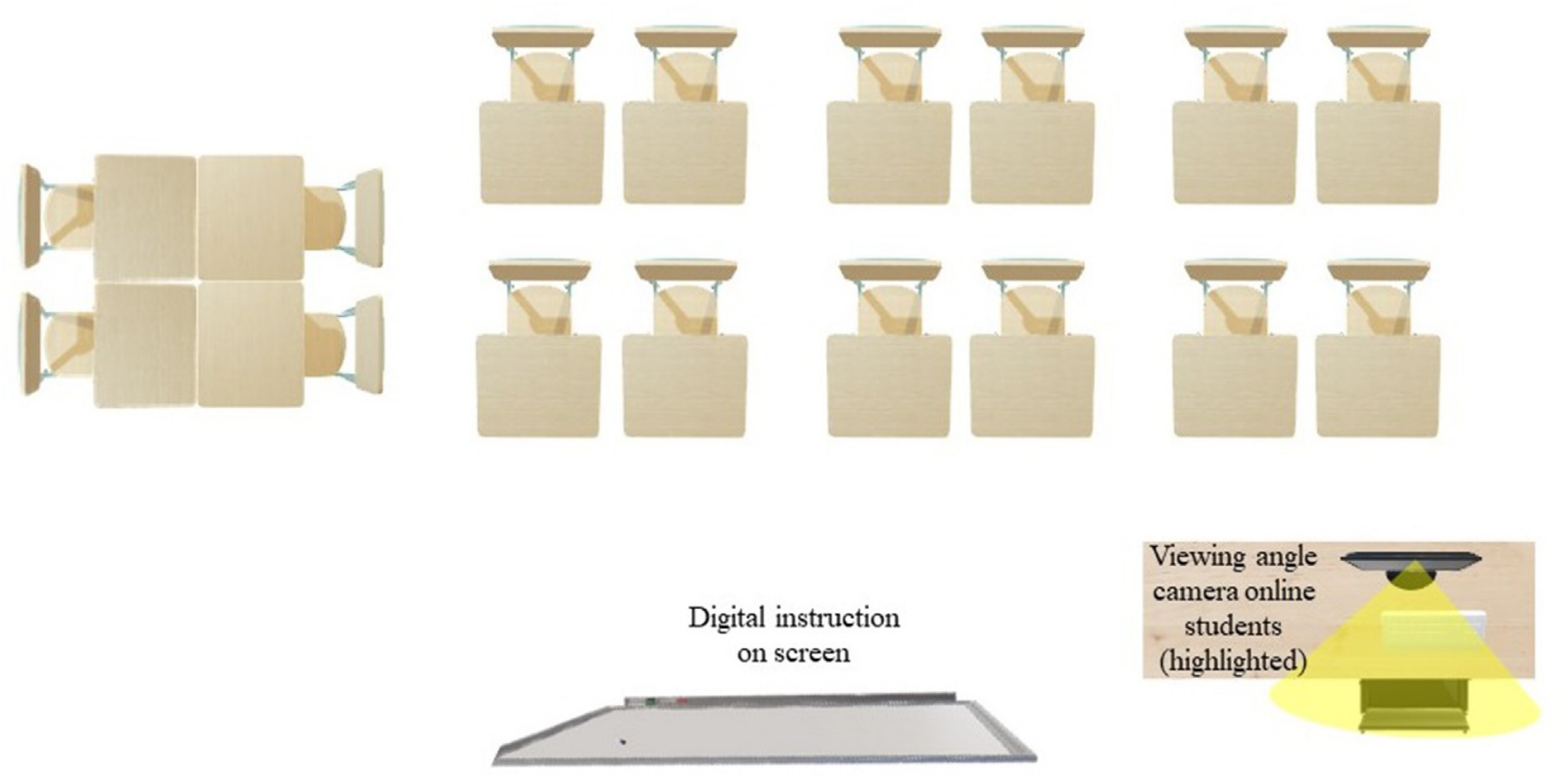
Example of a room set-up of Hybrid Virtual Classroom. The figure shows that the camera is at a fixed position.

First, test runs were conducted with existing available resources, including computers, screens with built-in cameras, microphones, and beamers to determine the optimal conditions for sound and camera position. Second, teachers were offered the opportunity to get acquainted with HVCs in a *test HVC*. This classroom included all resources and the actual setup (see Figure 1).

During the first weeks of the pilot, several extra ICT colleagues supported teachers who experienced technical problems. Additionally, student assistants were available to help starting classroom meetings and to moderate, especially the online learners, during the meeting, e.g. by monitoring incoming questions.

#### 3.2.2 Pedagogical support

Teaching in HVCs requires different pedagogical knowledge and skills (Raes et al., 2020b). Teachers had to simultaneously teach online and onsite students. Within the HEI, few teachers had experience with this way of teaching. Therefore, additional support was offered to guide and inspire teachers.

a. A video showing all the ins and outs of how to teach both groups at the same time;
b. A practical guide with pedagogical examples about how to teach in HVCs;
c. Various handouts with quick pedagogical and technical tips tailored to the different phases in a lesson;
d. A weekly quick and easy-to-apply tip for teachers to use in their daily classroom;
e. Kickstart meetings with the aim of informing, enthusing, and motivating teachers;
f. A weekly evaluation where teachers could share their experiences and for answering questions.

#### 3.2.3 Organizational support

Students of the part-time faculty could decide whether they wanted to attend classes online, onsite or alternately online and onsite. Groups were composed based on an inventory that was organized every ten weeks. After the first ten weeks, the group that wanted to follow all classes onsite had become smaller, and therefore could follow onsite education on a weekly basis. Furthermore, organizational support focused on the availability and presence of technical facilities in the classrooms and support staff.

## 4 Method

As stated before, HVCs were initialized as a pilot project aiming to improve student connectedness and being part of a community during the COVID-19 pandemic. To gain a better understanding of how students experience the feeling of a community of inquiry and how teachers try to enhance this feeling, two questions guided the study.

1. How do part-time students experience their social, teaching, and cognitive presence within a hybrid virtual classroom?
2. How do teachers stimulate social, teaching, and cognitive presence within a hybrid virtual classroom?

### 4.1 Material and methods

A mixed-method study was used for answering the research questions. Prior to conducting the research, the researchers followed the decision tree as formulated by the Saxion Ethical Advisory Committee. Given the nature of the study and the age of the participants, no additional clearance was required.

For selecting cases criteria selection was applied (Patton, 1987). First, teachers were selected who offered courses in HVCs during the first and second period. These teachers received a self-efficacy questionnaire to determine their perceptions of teaching in HVCs. These insights were used for selecting cases for this study. Second, only courses were selected which were identified by the head of quality assurance as ‘designed according to the educational rationale’. Selected courses had a student-driven approach and were designed according to a flipped classroom model. Therefore, these courses, corresponding teachers and students provided better insights into whether HVC as a pedagogical approach is useful.

For answering research question 1 quantitative data were collected by using the CoI questionnaire (Swan et al., 2008) among part-time students who attended one of the selected HVC-courses. The translated questionnaire by Eeckhout (2018) was used and adapted to fit the HEI’s part-time educational model (e.g., learning goals were changed into learning outcomes). The questionnaire consisted of 37 items and all items were measured using a 5-point Likert scale. Each of the three constructs of CoI was measured in the questionnaire. The questionnaire included items such as *The instructor was helpful in guiding the class towards understanding course topics in a way that helped me clarify my thinking (TP), I felt comfortable participating in the course discussions (SP) and I felt motivated to explore content-related questions (CP).* Given the aim of the study no further descriptive variables were collected.

For answering research question 2, qualitative data were collected through semi-structured interviews with teachers. The interviews focused on how teachers prepared and enacted in HVCs in relation to the CoI-model. The interview started with a sentence to be completed by the respondents: ‘*HVC result in…*’. Based on their answers and corresponding clarification follow-up questions and answers could be better related to the teachers' perceptions of HVCs. Other questions addressed how teachers prepared the lessons for teaching in HVCs, what they did to foster interaction between online and onsite groups, and how they experienced their own role. Due to COVID-19 restrictions, all interviews were conducted online. The interviews were recorded and transcribed.

In total 39 questionnaires were completed, and 9 interviews with teachers were conducted. For all participants, an informed consent was included in which the procedure, the form of data collection, topic of the study, involved research group, data management, privacy, and opportunities how to withdraw from the study were clarified. Prior to the collection of data, all participants filled in the informed consent.

#### 4.1.1 Participants

The participants of this study consisted of teachers and students. A total of 11 teachers were approached to take part in this study. Two teachers from the domain of Health Care declined, since they did not want to be involved in a research activity or felt that the classroom safety might be affected due to research activities. The pseudonyms of the participants and domains they work in are presented in Table 1.

**Table 1:** Overview of participants in the study.

Pseudonym	Domain
Mary	Health Care
Megan	Health Care
Sarah	Economics
Elisabeth	Economics
Harry	Economics
Jack	Economics
Samantha	Economics
William	Economics
John	Economics

The participating teachers offered a total of 13 courses. All students who attained one of these 13 courses received an invitation to take part in the study by filling out the CoI-questionnaire. Part-time students are 18 years or older and have a relevant workplace which is a prerequisite for the assignments. In total 173 students were invited to participate in the study. Out of the 39 students who started the questionnaire, 35 completed it. Given the number of (digital) evaluations that took place during the COVID-19 period, a reasonable number.

### 4.2 Data analysis

Quantitative data analysis started by calculating Cronbach’s alpha for the overall questionnaire and for teaching, social and cognitive presence. Additionally, Cronbach’s alpha for the whole concept of Community of Inquiry was determined. One item was removed prior to calculating Cronbach’s Alpha since it was not aligned with the original scale. As Table 2 shows, Cronbach’s alphas were high, indicating a reliable measure was used (DeVellis, 2003).

**Table 2:** Reliability of the scales.

Scale	Number of items	Cronbach’s alpha
CoI	36	.95
Teaching presence	14	.94
Social presence	10	.92
Cognitive presence	12	.90

After calculating Cronbach’s alpha, descriptive statistics (means and standard deviations) were calculated. Scores above 3.50 were identified as positive and scores below 2.50 as negative. After identifying means and standard deviations per scale, additional item analyses were included to identify highs and lows.

The qualitative data were analyzed by selecting meaningful quotes from the transcriptions, which were deductively coded. Two-level coding was applied. First, the type of presence for each quotation was determined. Second, it was determined whether the quotation referred to a 1. Experience, 2. Design decision, or 3. Tip/suggestion. Codes from both levels were applied to the quotations. After coding all interviews, experience(s), and design decision(s) were combined to reflect teachers' approach to stimulate social, teaching, and cognitive presences.

The first step of the coding process consisted of applying the codes to two interviews. Both interviews were coded by two of the authors, who independently coded the interview and then discussed similarities and differences. The second step was that insights based on the first coding experiences were shared in a meeting with all four authors. During this step, the code description and its application were further discussed. Once consensus was achieved, the subsequent interviews were divided among the authors. The third step was data reduction, in which quotes from the same code were tabulated per interview. This also resulted in some discussions about the application of codes and changes were only made if consensus was achieved between the authors. Table 3 shows the coding scheme.

**Table 3:** Overview of the coding scheme including example quotes.

Level	Code	Code description	Example quote
1	Social presence	Experiences related to social presence	Please turn on your camera when asking a question so that the group can see you.
1	Teaching presence	Experiences related to teaching presence	And then I keep talking behind such a screen, therefore I sometimes step aside, not visible for the [online] students, but still audible.
1	Cognitive presence	Experiences related to cognitive presence	That’s a course in which much is asked from the student in advance […] And as long as the students all do their preparation, there’s an opportunity for more depth. Then practical examples are shared and interpretation develops.
2	Experience	Relates to the experience of teaching in an HVC	Afterward, it turned out that form wasn’t always ideal for hybrid education, […]
2	Design	Relates to design decision prior to teaching in an HVC	Well, what I did is intentionally appoint an observer in the online group, so that the student was responsible for the conversation.
2	Tips/suggestion	Relates to tips/suggestions for teaching in an HVC	I’ll turn around the monitor so that they can see each other.

## 5 Results

### 5.1 Students' experienced social, cognitive, and teaching presence

**Figure 2:**
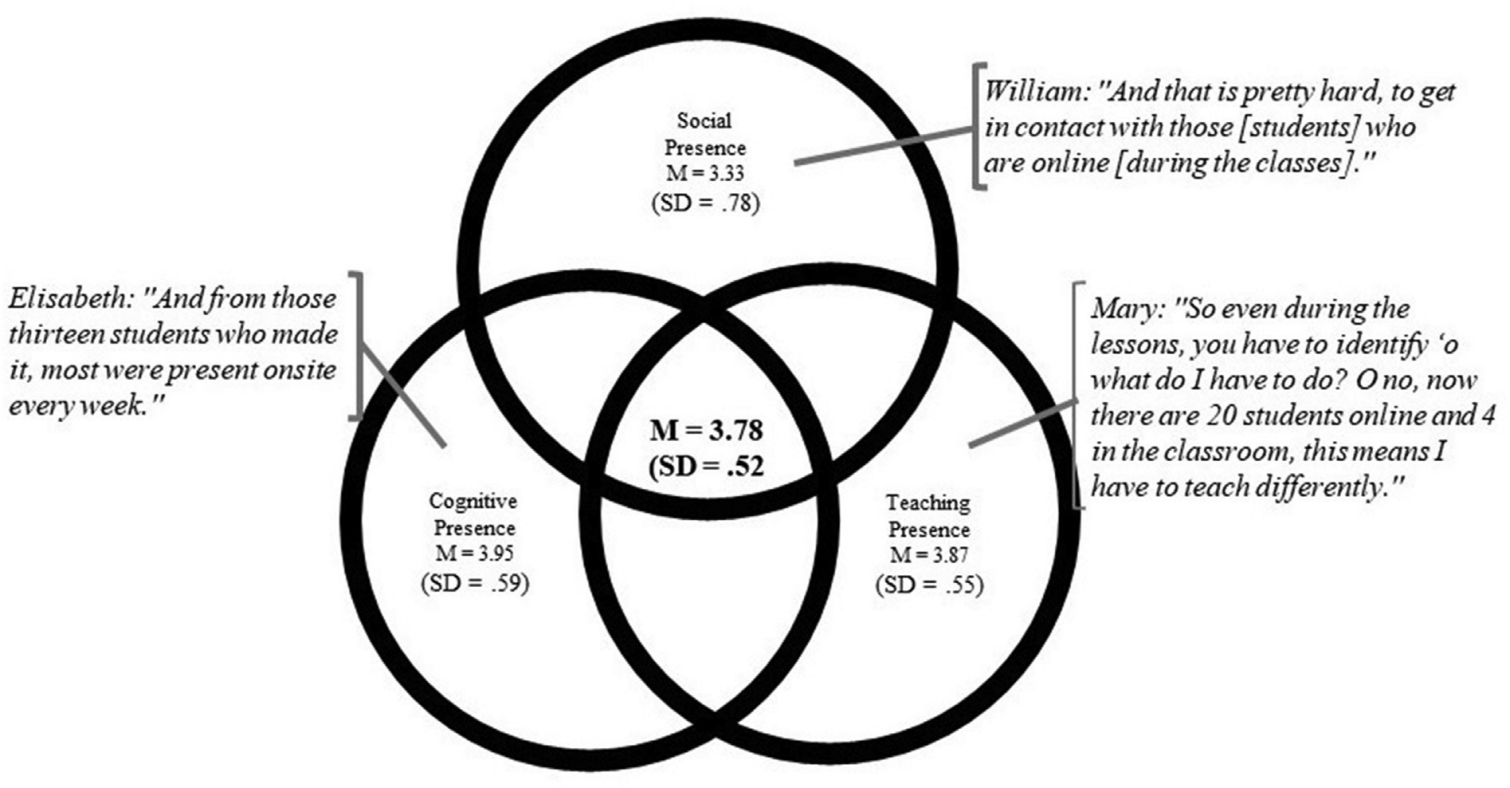
The CoI Framework including student experiences and quotes for teachers. The figure shows a quote for each presences and the means and standard deviations of all presences and CoI.

In general, students reported the perceived Community of Inquiry feeling as positive (M = 3.78; SD = .52). For each of the three presences, scores were neutral to positive, with low standard deviations (see Table 4 and Figure 2).

**Table 4:** Means and standard deviations for the presences.

Scale	Mean	Standard deviation
Social presence	3.33	.78
Teaching presence	3.87	.55
Cognitive presence	3.95	.59

The social presence scale was experienced as neutral by students. At item level (see Table 5), the items ‘*I felt comfortable interacting with other students through the ELO*’ (M = 3.05; SD = 1.03) and ‘*I felt comfortable conversating through the ELO*’ (M = 3.14; SD = 1.16) scored relatively low. Students scored higher on the items ‘*Discussions help me to develop a sense of collaboration*’ (M = 3.54; SD = .93) and ‘*I felt that my point of view was acknowledged by other students*’ (M = 3.73; SD = .77).

**Table 5:** Means and standard deviations for items within the social presence scale.

Item	N	M	SD
Getting to know other course participants gave me a sense of belonging in the course.	37	3.41	1.19
I was able to form distinct impressions of some course participants.	37	3.24	1.14
Online communication through Blackboard Collaborate is an excellent medium for social interaction.	37	3.14	1.16
I felt comfortable conversing through the ELO (online medium.)	37	3.14	1.16
I felt comfortable participating in collaborative learning activities	37	3.27	1.05
I felt comfortable interacting with other course participants.	37	3.05	1.03
I felt comfortable disagreeing with other course participants while still maintaining a sense of trust.	37	3.32	.97
I felt that, when I attended the course online, I had sufficient opportunities to express my opinion	37	3.43	1.02
I felt that my point of view was acknowledged by other course participants.	37	3.73	.77
Discussions help me to develop a sense of collaboration.	37	3.54	.93

Within the teaching presence scale (see Table 6), all mean scores were above 3.72. The item with the 3.72 score addressed whether teachers encouraged students to explore new concepts in the courses. The highest score within the teaching presence scale related to the clear communication of the teacher about the due dates/time frames for learning activities.

**Table 6:** Means and standard deviations for items within the teaching presence scale*.*

Item	N	M	SD
The instructor clearly communicated important course topics	39	4.03	.74
The instructor clearly communicated important course goals	39	4.13	.66
The instructor provided clear instructions on how to participate in course learning activities.	39	3.92	.74
The instructor clearly communicated important due dates/time frames for learning activities.	39	4.23	.74
The instructor was helpful in identifying areas of agreement and disagreement on course topics that helped me to learn.	39	3.92	.74
The instructor was helpful in guiding the class towards understanding course topics in a way that helped me clarify my thinking.	39	4.03	.74
The instructor helped to keep *online* course participants engaged and participating in productive dialogue.	39	3.90	.94
The instructor helped to keep *onsite* course participants engaged and participating in productive dialogue.	39	3.85	.96
The instructor helped keep the course participants on task in a way that helped me to learn.	39	4.08	.62
The instructor encouraged course participants to explore new concepts in this course.	39	3.72	.89
Instructor actions reinforced the development of a sense of community among course participants.	39	3.85	.78
The instructor helped to focus discussion on relevant issues in a way that helped me to learn.	39	3.79	.73
The instructor provided feedback that helped me understand my strengths and weaknesses relative to the course’s goals and objectives.	39	3.87	.98
The instructor provided feedback in a timely fashion.	39	3.97	.84

The cognitive presence scale (see Table 7) revealed that students expect that they can apply the knowledge created in their profession (M = 4.26; SD = .56) and that learning activities enhanced students' curiosity (M = 4.03; SD = .71). Within the scale, the items ‘*learning activities helped me construct explanations/solutions*’ (M = 3.49; SD = .89) and ‘*reflection on course content and discussions helped me understand fundamental concepts in this module*’ (M = 3.57; SD = 1.04) scored relatively low.

**Table 7:** Means and standard deviations for items within the cognitive presence scale*.*

Item	N	M	SD
Problems posed increased my interest in course issues.	35	3.89	.96
Course activities piqued my curiosity.	35	4.03	.71
I felt motivated to explore content-related questions	35	4.03	.71
I utilized a variety of information sources to explore problems posed in this course.	35	4.03	.75
Brainstorming and finding relevant information helped me resolve content-related questions	35	3.94	.77
Online discussions were valuable in helping me appreciate different perspectives	35	3.74	.78
Combining new information helped me answer questions raised in course activities	35	3.77	.69
Learning activities helped me construct explanations/solutions.	35	3.49	.89
Reflection on course content and discussions helped me understand fundamental concepts in this class.	35	3.57	1.04
I can describe ways to test and apply the knowledge created in this course.	35	3.94	.73
I have developed solutions to course problems that can be applied in practice.	35	3.74	.95
I can apply the knowledge created in this course to my work or other non-class related activities.	35	4.26	.56

### 5.2 Teachers' stimulation of social, teaching, and cognitive presence

The HVCs were introduced to enhance the feeling of the social presence of students during the COVID-19 period. In the interviews, teachers reported both their experiences and their deliberate decisions to enhance social, cognitive, and teaching presence (see Figure 2).

#### 5.2.1 Social presence

When teachers prepared their lessons, they had little insights into which students attended classes online and whom onsite. As Samantha illustrated “*However, every time it is a surprise and who do you meet as a teacher [onsite] and which students you meet [onsite] and how many [students] you meet [onsite].*” According to teachers, this affected their teaching methods, since the number of students per location influences which teaching methods are relevant to enhance students' social presence. As Harry, Elisabeth, and Megan experienced, the variety in students per location affected how they included the students. William explained that enforcing interaction between both groups is hard and clarified this as follows: “*And that is pretty hard, to get in contact with those [students] who are online [during the classes]. It is essentially becoming one-way traffic*”. In general, five teachers experienced that involving the online students is more challenging than those onsite, despite that they do their best to engage all students (Samantha and William). As Sarah, William, Harry, John, and Elisabeth noticed, they experienced various difficulties with enhancing social presence. For example, Sarah and Samantha indicated that online students are less connected during the lessons, which affects the classroom dynamics. Harry also indicated that he found it more challenging to include the online group: “*[… I] have to pay more attention [to them], than the online group is actively involved. Actively involving them in the lesson.*” Their experiences also made them more aware of the role they have as a teacher in HVCs, whereas Mary sees herself as a moderator and coach to achieve deeper learning, John finds himself as a facilitator to facilitate student interaction.

In addition to their own role, teachers experienced that (learning) activities influenced the social presence. Whereas Samantha included physical tea meetings to enhance social interaction, Elisabeth changed the camera position to the persons who were speaking. Sarah asked the online students to turn on their cameras to foster social connectedness, especially at the moments that interaction between groups was needed. Finally, John included learning activities in which he encouraged all students to pose questions.

As Sarah, Mary and Elisabeth identified a prerequisite for social presence is that webcam of the online students is turned on. Mary and Sarah pointed out that this is only possible if a safe learning environment is created. This feeling of a safe learning environment is negatively affected, according to Elisabeth, if the online students are considered observers rather than peer-learners.

#### 5.2.2 Teaching presence

Prior to teaching in HVCs, teachers had limited time to (re)design their course to suit HVCs. Sarah and William did not adapt their course design to suit the HVC. In contrast, Samantha and Mary prepared each lesson separately to make it possible to adapt it to the specific division of students onsite and online and their own experiences. Also, Megan, Sarah, and Jack adapted their teaching methods to suit their lessons to teach both online and onsite students. Consequently, teachers included more tools or alternative learning activities in their lessons, such as polls (Samantha and William), whiteboard brainstorming tools (Samantha, William, and Elisabeth), and online quizzes (Sarah, Jack, and John). Based on their experiences, teachers also saw opportunities for the long term, including applying online games with foreign students (Sarah) or new ways to organize classroom meetings (William).

At the start of teaching in HVCs, Harry, Mary, Elisabeth, and John experienced that they felt like starting teaching professionals again. According to them, teaching in HVCs was (completely) different from their regular teaching. Mary experienced more challenges with classroom management, including monitoring the chat while teaching. Elisabeth, Harry, and Mary noted that hybrid teaching required more energy than physical teaching. This can also be the result of teachers not knowing beforehand how the allocation of online and onsite students was. As Mary illustrated “[…] *you don*’*t know who is onsite and how is online […] Hence, chaos can be emerging, since lesson materials do not adequately suit the reality. So even during the lessons, you have to identify* ‘*o what do I have to do? O no, now there are 20 students online and 4 in the classroom, this means I have to teach differently.*”

While offering the HVCs, teachers argued that they focused on the largest group of students, despite that they wanted to divide attention to both groups (William, Harry, Mary, Elisabeth, and Megan). They indicated that this might also be the most challenging aspect of teaching in HVCs. William explained: “*The main point is, what I catch myself doing, is in a large group, I*’*m more focused on what is happening in the group of students who are onsite.*” Additionally, Sarah, William, Harry, and John argued that the lack of non-verbal communication was also an issue, since they only received signals from students fragmentarily. To prevent that students might miss important information, teachers felt that they had to be clearer in communication and identifying expectations (Elisabeth and Megan). Sarah, William, Elisabeth, and Megan argued that they stimulated communication and interaction between the online and onsite group, for example by posing questions to both groups (Sarah and Megan) or by giving turns to specific students (Elisabeth). Despite this awareness, William and Elisabeth still found it challenging to engage students, since this requires, according to them, extensive pedagogical skills. Samantha, Elisabeth, Jack, and Megan all indicated their actions as a teacher were decisive in the way students behaved. But according to Elisabeth, Megan and Jack students also influenced teacher behavior.

Finally, Samantha and Elisabeth experienced that their classroom movement was reduced by the camera position. Elisabeth provided several examples of how she tried to overcome this issue while teaching, including moving the camera to other positions to focus on either the whiteboard or the onsite group.

#### 5.2.3 Cognitive presence

From all three presences, cognitive presence is least mentioned by teachers. Teachers found it challenging when students came to class unprepared. Megan specified “*[…something] I*’*ve been stricter on: it*’*s your course and when you*’*re not prepared then it*’*s hard to follow my lessons*”*.* William found it challenging to motivate students. Jack emphasized that establishing clear relationships between the instruction, the learning activities, and the assessment criteria is important to ensure student engagement.

Teachers indicated their instructions in the hybrid classroom are concise and shorter. As a result, Elisabeth and John found it difficult to deepen instruction. Elisabeth thought students learned less from her in the HVC setting because it is difficult to add side steps in your instruction. She said: “*So normally I deviate every now and then, but now I*’*m more concerned with, and that*’*s something I don*’*t really like, but I*’*m more concerned with getting them to the finish line*”. Because of the setting, the teacher is forced to give linear, to-the-point instruction. Sarah, William, and Elisabeth also experienced more difficulties, especially with regard to the online students, in checking students' understanding of the subject matter and the progress in their learning process.

According to John and Jack, the teacher has to take deliberate action in an HVC to initiate and create the preconditions for dialogue among students. Jack illustrated: “*Discussions don*’*t arise spontaneously*”. Megan named it as “*directing action to engage students and get them to work*”. Especially when it comes to mutual interaction between the student groups online and onsite more teaching skills are required than in a fully onsite setting. Jack, Megan, and John stated that as a teacher it is important to divide your attention across both groups and ask reflective questions that deepen and strengthen the interaction. Megan applied a teaching method in which interaction among students was necessary: “*[…], because there were peer groups within which students had to give feedback on each other*’*s videos. That was also part of the grading rubric, that your feedback from peers has a part in your own reflection.*”

It is not possible to give an unequivocal answer to the question of whether the quality of the delivered professional products has deteriorated or remained the same. Elisabeth and Mary indicated the knowledge transfer among students who attended classes onsite is greater than students who participated online. Elisabeth found it interesting that*:* “*Thirteen [from twenty] students submitted a professional product [at the end of the quartile]. And from those thirteen students who made it, most were present onsite every week*”. As a result of the corona pandemic and the related circumstances, Elisabeth’s students were not always able to fully complete their assignments within their work context. Mary, John and Megan found it difficult to teach skills to online students and provide them with feedback. Mary indicated many of the freshman students were failing with regard to basic conversation skills. Whether this is caused by the hybrid classroom cannot be said for sure. She added that students made their own choice in attending class onsite or online.

### 5.3 Teachers' suggestions for improving COI

During the interviews, teachers also reflected on their experiences and looked forward to how they could tackle the experienced problems. They provided also tips for fellow teachers regarding teaching and social presence. Various tips addressed strengthening the interaction between the online and onsite students. At the pedagogical level, Elisabeth, Mary, and Harry suggested learning activities to deepen the interaction between students.

Tips have also been given in the field of teaching presence regarding technical support. For example, teachers advised to first prefer technical support over pedagogical support. Elisabeth emphasized the importance of a small-scale approach by first ensuring that the technology is fully operational in a number of classrooms. If so, expansion to other classrooms is possible. Five teachers emphasized the importance of sparring with fellow teachers and exchanging experiences. For example, Mary gave the tip to be open and to discuss things if something doesn’t turn out as planned: “*First of all, keep calm, be sincere and discuss what challenge you are facing... make it discussable.*”

Three teachers expressed the need for additional cameras in the HVCs (Megan, Mary, and Harry), since this might ensure optimization of interaction between groups and provide students freedom of movement. John and Elisabeth indicated that additional insights are needed on what the optimal group size is for both online and onsite groups.

## 6 Discussion

During the COVID-19 pandemic, several new ways of teaching and learning were introduced to continue education within legislation (Sunitha et al., 2022; Triyason et al., 2020). Often new technologies were introduced in a short period of time and both teachers and students needed to adapt to the new teaching and learning approaches (Vital-López et al., 2022). Whereas in the beginning of the pandemic the focus was on full online education, other forms of education were introduced around the summer of 2020. In this study, piloting with HVCs in higher education illustrated the potential for both online and onsite students. Whereas HVCs come in various designs, this study addressed the implementation of synchronous HVCs as a means to enhance students' social connectedness. As recent studies illustrate (e.g., Butnaru et al., 2021; Collazos et al., 2021; Shi et al., 2021) social connectedness is a prerequisite for students to attend lessons and be cognitively engaged. It requires interaction and well-regulated student awareness and emotions within HVCs, which is particularly challenging in online learning. Although our study showed the potential of HVCs for enhancing students' feeling of CoI, students' social presence can be further enhanced. Technical limitations and issues might have affected to what extent students felt connected with their peers. Additionally, an issue with (partly) online education is that not all learners turned on their camera. This affects social connectedness, since students are less visible to one another. Consequently, they might become a spectator of the course, instead of a student. To overcome this challenge, teachers need to apply various strategies to include both groups (Meer et al., 2021).

In addition, in order to facilitate learning in HVCs, teachers need to offer courses aligned with their own teaching preferences or need support to provide courses as expected (Borko, 2004). As this study demonstrated, due to the technical set-up of HVCs, teachers felt limited in their movement and, therefore, their lessons became more static than normal. Teachers that were interviewed in the study of Raes (2022) mentioned the possibility to naturally move around and use the whiteboard and the fact that they could see the online students on screens as important advantages of the HVC set-up in their institution. In general, creating opportunities for teacher movement in classrooms seems to have a promising impact on instructional practices and student learning (Talbert and Mor-Avi, 2019) So, overcoming these technical limitations in the HVC set up within the HEI involved in this study and enabling more freedom of movement for teachers could be a way to further enhance teaching in HVCs.

As a result of the COVID-19 period, teachers of all involved courses were asked to offer their courses in HVCs. However, as the results of this study demonstrated, it can be argued whether all courses are suitable within the HVC-context. For example, in courses in which skill development (e.g. conversation skills, coaching skills) and classroom safety are vital aspects, offering courses in HVCs might reduce students' active involvement in classroom activities. They might feel observed and uncertain whether screen captures are made of their involvement. Additionally, other course-specific goals might also reduce the effectiveness of HVCs. Therefore, when determining if HVCs are introduced, decisions need to be made for which content and with what aim. Once these decisions are made, organizational support can be started. On the one hand, this addresses the onsite technical materials and teacher preparation, on the other hand, organizational support also provides insights into how many students attend classes online and onsite. These latter insights help teachers to determine which learning activities can be offered during the lessons.

Besides the matter of suitability of courses and their learning outcomes, it can also be questioned whether all teachers sufficiently meet the demands that are required for teaching in HVCs. This study shows that teaching in an HVC is very demanding. Training and support for teachers, both pedagogically and technologically, is very desirable (Fernández-Batanero et al., 2022; Raes et al., 2020a; Triyason et al., 2020). Teachers need additional skills to their expertise built on training and experience in a traditional onsite setting (Koehler and Mishra, 2009). And maybe even more fundamental, their beliefs and attitudes with respect to good education and its design should match the principles of teaching in an HVC. The teachers in this study had a relatively positive attitude toward the concept of HVC, but changing beliefs often is a long process that should be taken into account when implementing rigorous changes in educational designs (Borko, 2004).

The HVC certainly has its potential when it comes to enhancing flexibility. It offers students a choice regarding the location from which they attend their classes. This makes courses more accessible to a broader group of (potential) students. Especially in the context of part-time education in which this study was conducted.

## 7 Limitations

The results of this study should be interpreted in the context of some defining characteristics of the research design. To start with, a mixed-method approach among two distinct target groups provided triangulation and complementary insights. Participants were not randomly selected. The selection of teachers for interviews was based on a combination of a self-report questionnaire and perceived characteristics of the course design. This might have resulted in the selection of relatively enthusiastic teachers regarding the HVC concept. Furthermore, more teachers from courses in the economic domain than the health care domain were included, which might affect generalizability of the study.

Second, students were voluntarily invited to fill in the CoI-questionnaire. As a result of the amount of questionnaires and evaluations that were disseminated during the pandemic, response rates were low. Within the questionnaire, no additional background variables were included, since the leading research question does not focus on differences between, for example, age groups, gender and cultural backgrounds. However, this makes the interpretation, especially with regard to students' technical skills, more difficult. Additionally, the CoI-questionnaire is a validated instrument, which uses a self-report measure. This might also affect how students score the questionnaires. Furthermore, only quantitative data were collected from the students. As a result, relevant aspects of the context of the given answers might have been missed by the researchers. Problems encountered in this study were partly related to the Internet speed and the minimum available technical capabilities in which the standard classrooms were adapted. This stresses the importance of conscious consideration of whether elements of the HVC perceived positively in this study also seem promising in one’s own context before moving on to implementation.

## 8 Conclusions and suggestions

Overall students felt positive about HVCs, which was showed by the feeling of being part of a CoI. Within the presences, the social presence was experienced most neutral by students. Based on the results of social presence, it can be concluded that optimizing the used technology is expected to contribute to more connectedness among students.

Teachers also felt positive about teaching in an HVC. Teachers apply many interventions both consciously and unconsciously to promote each presence. However, they did experience some limitations related to the technical possibilities. Technology at that time was not yet sufficient to meet all the requirements that HVCs entail. Due to the fixed position of the camera, there was limited freedom of movement for teachers. It also made it more difficult to promote interaction between both groups and keep online students fully engaged. They recommended an extra screen so that they could see all the online students and still see the presentation or assignment on another screen. That would also make it easier for them to treat all participating students. Teachers now tend to treat them separately.

The results of both teachers and students show that hybrid virtual classrooms can offer added value in making education more flexible. To unleash the full potential of HVCs, additional insights are needed on how to optimize the interaction between the online and onsite group and what support and technical resources are needed to support teaching. It also seems relevant to further investigate what kind of course content and intended learning outcomes are more or less suitable for teaching in an HVC.

## Declarations

### Author contribution statement

Tjark Huizinga; Rosalien van der Meer: Conceived and designed the experiments; Performed the experiments; Analyzed and interpreted the data; Contributed reagents, materials, analysis tools or data; Wrote the paper.

Anne Lohuis; Judith Zwerver – Bergman: Conceived and designed the experiments; Performed the experiments; Analyzed and interpreted the data; Wrote the paper.

### Funding statement

This research did not receive any specific grant from funding agencies in the public, commercial, or not-for-profit sectors.

### Data availability statement

Data will be made available on request.

### Declaration of interest’s statement

The authors declare no competing interests.

### Additional information

No additional information is available for this paper.

## Appendix A Supplementary data

Questionnaire CoI - blinded Questionnaire CoI - blinded

## References

[bib1] Anderson T., Rourke L., Garrison D.R., Archer W. (2001). Assessing teaching presence in a computer conferencing context. J. Async. Learn. Netw..

[bib2] Borko H. (2004). Professional development and teacher learning: mapping the terrain. Educ. Res..

[bib3] Bower M., Dalgarno B., Kennedy G.E., Lee M.J.W., Kenney J. (2015). Design and implementation factors in blended synchronous learning environments: outcomes from a cross-case analysis. Comput. Educ..

[bib4] Butnaru G.I., Haller A., Dragolea L., Anichiti A., Tacu Hârsan G. (2021). Students’ wellbeing during transition from onsite to online education: are there risks arising from social isolation?. Int. J. Environ. Res. Publ. Health.

[bib5] Castellanos-Reyes D. (2020). 20 years of the community of inquiry framework. TechTrends.

[bib6] Chen R.H. (2022). Effects of deliberate practice on blended learning sustainability: a community of inquiry perspective. Sustain. Times.

[bib7] Cohen A., Holstein S. (2018). Analysing successful massive open online courses using the community of inquiry model as perceived by students. J. Comput. Assist. Learn..

[bib8] Collazos C.A., Fardoun H., AlSekait D., Pereira C.S., Moreira F. (2021). Designing online platforms supporting emotions and awareness. Electron.

[bib9] Desimone L.M. (2009). Improving impact studies of teachers’ professional development: toward better conceptualizations and measures. Educ. Res..

[bib10] DeVellis R.F. (2003).

[bib11] Eeckhout A. (2018).

[bib12] Fernández-Batanero J.M., Montenegro-Rueda M., Fernández-Cerero J., Tadeu P. (2022). Online education in higher education: emerging solutions in crisis times. Heliyon.

[bib13] Garrison D.R. (2007). Online community of inquiry review: social, cognitive, and teaching presence issues. J. Async. Learn. Network.

[bib14] Garrison D.R. (2011).

[bib15] Garrison D.R. (2017).

[bib16] Garrison D.R., Anderson T., Archer W. (2000). Critical inquiry in a text-based environment: computer conferencing in higher education. Internet High Educ..

[bib17] Garrison D.R., Anderson T., Archer W. (2001). Critical thinking, cognitive presence, and computer conferencing in distance education. Am. J. Dist. Educ..

[bib18] Hilliard L.P., Stewart M.K. (2019). Time well spent: creating a community of inquiry in blended first-year writing courses. Internet High Educ..

[bib20] Kalmar E., Aarts T., Bosman E., Ford C., Kluijver L. de, Beets J., Veldkamp L., Timmers P., Besseling D., Koopman J., Fan C., Berrevoets E., Trotsenburg M., Maton L., Remundt J., van, Sari E., Omar L., Beinema E., Winkel R., Sanden M. van der (2022). The covid-19 paradox of online collaborative education: when you cannot physically meet, you need more social interactions. Heliyon.

[bib21] Kaul M., Aksela M., Wu X. (2018). Dynamics of the community of inquiry (CoI) within a massive open online course (MOOC) for in-service teachers in environmental education. Educ. Sci..

[bib22] Koehler M.J., Mishra P. (2009). What is technological pedagogical content knowledge?. Contemp. Issues Technol. Teach. Educ..

[bib23] Le Roux I., Nagel L. (2018). Seeking the best blend for deep learning in a flipped classroom – viewing student perceptions through the Community of Inquiry lens. J. Educ. Technol. High. Educ..

[bib24] Lohuis A., Huizinga T., Mannetje J. 't, Visscher-Voerman I. (2021). De implementatie van het didactisch concept flipped classroom in het deeltijdonderwijs in het hbo. Een exploratieve casestudy. [The implementation of the didactical concept flipped classroom in part time higher professional education. An explorative case study]. Tijdschrift Hoger Onderwijs.

[bib25] Lohuis A., Huizinga T., Slaghuis J., Zwerver J., Meer R. van der, Engelhart M. (2021). https://blogs.gre.ac.uk/learning-teaching/2021/10/04/preparing-teachers-for-teaching-in-hybrid-virtual-classrooms/.

[bib26] Meer R. van der, Oijen J. van, Venema A., Oosterwijk R. (2021). https://www.versnellingsplan.nl/wp-content/uploads/2022/06/Handreiking-sociale-binding-online-blended-leergemeenschappen.pdf.

[bib27] Molano S., Polo A. (2015). Social network analysis in learning community. Procedia – Soc. and Behav. Sci..

[bib28] Osguthorpe R., Graham C. (2003). Blended learning environments: definitions and directions. Q. Rev. Dist. Educ..

[bib29] Patton M.Q. (1987).

[bib30] Raes A. (2022). Exploring student and teacher experiences in hybrid learning environments: does presence matter?. Postdigital Sci. Educ..

[bib31] Raes A., Detienne L., Windey I., Depaepe F. (2020). A systematic literature review on synchronous hybrid learning: gaps identified. Learn. Environ. Res..

[bib32] Raes A., Vanneste P., Pieters M., Windey In, Van den Noortgate W., Depaepe F. (2020). Learning and instruction in the hybrid virtual classroom: An investigation of students’ engagement and the effect of quizzes. Comput. Educ..

[bib33] Shea P., Bidjerano T. (2009). Community of inquiry as a theoretical framework to foster “epistemic engagement” and “cognitive presence” in online education. Comput. Educ..

[bib34] Shea P., Hayes S., Vickers J., Gozza-Cohen M., Uzuner S., Mehta R., Valchova A., Rangan P. (2010). A re-examination of the community of inquiry framework: social network and content analysis. Internet High Educ..

[bib35] Shea P., Richardson J., Swan K. (2022). Building bridges to advance the Community of Inquiry framework for online learning. Educ. Psychol..

[bib36] Shi Y., Tong M., Long T. (2021). Investigating relationships among blended synchronous learning environments, students’ motivation, and cognitive engagement: a mixed methods study. Comput. Educ..

[bib37] Stenbom S. (2018). A systematic review of the Community of Inquiry survey. Internet High Educ..

[bib38] Sunitha P., Ahmad N., Barbhuiya R.K., Gunjan V.K., Ansari M.D., Kumar A., Mozar S. (2022). ICCCE 2021. Lecture Notes in Electrical Engineering.

[bib39] Swan K., Richardson J.C., Ice P., Garrisson D.R., Cleveland-Innes M., Arbaugh J.B. (2008). Validating a measurement tool of presence in online communities of inquiry. E-Mentor.

[bib40] Talbert R., Mor-Avi A. (2019). A space for learning: an analysis of research on active learning spaces. Heliyon.

[bib41] Triyason T., Tassanaviboon A., Kantharmanon P. (2020).

[bib42] Thymniou A., Tsitouridou M. (2021). Book: Research on E-Learning and ICT in Education.

[bib43] Vital-López L., García-García R., Rodríguez-Reséndíz J., Paredes-García W.J., Zamora-Antuñano M.A., Oluyomi-Elufisan T., Rodríguez Reséndiz H., Álvarez Sánchez A.R., Cruz-Pérez M.A. (2022). The impacts of COVID-19 on technological and polytechnic university teachers. Sustain. Times.

[bib44] Wang H. (2005). A qualitative exploration of the social interaction in an online learning community. Int. J. Technol. Teach. Learn..

