# Supplementary material for: Student and teacher perceptions of community of inquiry in hybrid virtual classrooms
Source: Heliyon. 2022 Dec 24;8(12):e12549. doi: 10.1016/j.heliyon.2022.e12549 (PMC9813719; doi:10.1016/j.heliyon.2022.e12549)
Supplement: Questionnaire CoI - blinded [file mmc1.docx]

Dear student,

Thank you for participating in this study regarding hybrid virtual classrooms. You will receive a questionnaire addressing your experiences about hybrid virtual classrooms, in which we focus on experience connectedness with both the content and your fellow students. Additional questions regarding the current way of teaching compared to the regular experiences (in which all students attend the lessons on location) are included.

Before you will enter the questionnaire, we are obliged to offer you an informed consent. In this informed consent we explain how data is handled. Additionally, you have the opportunity to exclude your data at any moment during the study. When you’ve got questions you can contact the head researcher.

When filling the questionnaire we ask you to share your opinion regarding the statements, there are no good or bad answers. By answering the statements, you will help to improve your own education and that of your fellow students.

All reports will be anonymized and, therefore, not be trackable to individual participants. Filling in the questionnaire takes approximately 15 minutes.

Kind regards

The research team.

**Informed consent**

In order to be able to use your research data, we ask your permission. Below, you will find a digital way to grant your permissions. The permission you will give has the same value as those on a signed informed consent. After approving the informed consent, you will get the questions.

I declare that I have sufficiently and clearly being informed about the nature and methods of this study. I voluntarily participate in this study and acknowledge that results of the questionnaire can be used for selecting purposes. My data will be anonymized. Personal information will not be transferred or used to external parties without my explicit approval.

I have understand previous text and I approve to cooperate in this study.

Data:

- I agree [go to questions]
- I disagree [go to end of survey]

**Questionnaire**

1. I’m enrolled as a [part-time student for a degree / student for a single course]
2. I attend the following degree [name educational program]
3. This questionnaire will be filled-in for the following course: [name course]
4. What amount of time did you attend the courses onsite/online for this course?
   - Onsite during even weeks
   - Onsite during uneven weeks
   - Completely online
   - Completely onsite
5. How many meetings of this course did you attend on location?
   1. (1-9)

Give your opinion (on a 5 point Likert scale totally disagree – totally agree) on the following statements. These statements relate both to the instructors communication during the course activities as via the electronic learning environment:

1. The instructor clearly communicated important course topics.
2. The instructor clearly communicated important course goals.
3. The instructor provided clear instructions on how to participate in course learning activities.
4. The instructor clearly communicated important due dates/time frames for learning activities.

Give your opinion (on a 5 point Likert scale totally disagree – totally agree) on the following statements. These statements are related to the course meetings (both onsite as online)

1. The instructor was helpful in identifying areas of agreement and disagreement on course topics that helped me to learn.
2. The instructor was helpful in guiding the class towards understanding course topics in a way that helped me clarify my thinking.
3. The instructor helped to keep online course participants engaged and participating in productive dialogue.
4. The instructor helped to keep onsite course participants engaged and participating in productive dialogue.
5. The instructor helped keep the course participants on task in a way that helped me to learn.
6. The instructor encouraged course participants to explore new concepts in this course.
7. Instructor actions reinforced the development of a sense of community among course participants.
8. The instructor helped to focus discussion on relevant issues in a way that helped me to learn.
9. The instructor provided feedback that helped me understand my strengths and weaknesses relative to the course’s goals and objectives.
10. The instructor provided feedback in a timely fashion.

Give your opinion regarding the following statements (on a 5 point Likert scale totally disagree – totally agree):

1. Getting to know other course participants gave me a sense of belonging in the course.
2. I was able to form distinct impressions of some course participants.
3. Online communication through Blackboard Collaborate is an excellent medium for social interaction.
4. I felt comfortable conversing through the ELO (online medium.).
5. I felt comfortable participating in collaborative learning activities.
6. I felt comfortable interacting with other course participants.
7. I felt comfortable disagreeing with other course participants while still maintaining a sense of trust.
8. I felt that, when I attended the course online, I had sufficient opportunities to express my opinion.
9. I felt that my point of view was acknowledged by other course participants.
10. Discussions help me to develop a sense of collaboration.

Give your opinion regarding the following statements (on a 5 point Likert scale totally disagree – totally agree):

1. Problems posed increased my interest in course issues.
2. Course activities piqued my curiosity.
3. I felt motivated to explore content-related questions.
4. I utilized a variety of information sources to explore problems posed in this course.
5. Brainstorming and finding relevant information helped me resolve content-related questions.
6. Online discussions were valuable in helping me appreciate different perspectives.
7. Combining new information helped me answer questions raised in course activities.
8. Learning activities helped me construct explanations/solutions.
9. Reflection on course content and discussions helped me understand fundamental concepts in this class.
10. I can describe ways to test and apply the knowledge created in this course.
11. I have developed solutions to course problems that can be applied in practice.
12. I can apply the knowledge created in this course to my work or other non-class related activities.
13. The teacher stimulated interaction between the online and onsite group of students.

I believe that the amount of interaction between the online and onsite students:

- Was far less than if we would have been on the same location.
- Was less than if we would have been on the same location.
- Was the same as if we would have been on the same location.
- Was more than if we would have been on the same location.
- Was far more than if we would have been on the same location.

I think that the extent to which the teacher promoted me to learn:

- Was far less than if I was able to attend all lessons on location.
- Was less than if I was able to attend all lessons on location.
- Was the same as if I was able to attend all lessons on location.
- Was more than if I was able to attend all lessons on location.
- Was far more than if I was able to attend all lessons on location.

I think that what I have learned during this course was:

- Was far less than if I was able to attend all lessons on location.
- Was less than if I was able to attend all lessons on location.
- Was the same as if I was able to attend all lessons on location.
- Was more than if I was able to attend all lessons on location.
- Was far more than if I was able to attend all lessons on location.

I think that the required motivation for learning, as a result of how the course was offered

- Was far less than if I was able to attend all lessons on location.
- Was less than if I was able to attend all lessons on location.
- Was the same as if I was able to attend all lessons on location.
- Was more than if I was able to attend all lessons on location.
- Was far more than if I was able to attend all lessons on location.

I think that attending lessons on location results in

- Far less motivation than if I would attend the lessons online.
- Less motivation than if I would attend the lessons online.
- Equal motivation than if I would attend the lessons online.
- Stronger motivation than if I would attend the lessons online.
- Far stronger motivation than if I would attend the lessons online.

I think that sharing experiences with fellow students and teachers on location outside the lessons results in:

- Far less motivation than if I would attend the lessons online.
- Less motivation than if I would attend the lessons online.
- Equal motivation than if I would attend the lessons online.
- Stronger motivation than if I would attend the lessons online.
- Far stronger motivation than if I would attend the lessons online.

I think that, as a result of exchanging experiences on location, the quality of my final product (assessment)

- Is far lower than if I would have exchanged experiences online.
- Is lower than if I would have exchanged experiences online.
- Is equal to what I would have exchanged experiences online.
- Is higher than if I would have exchanged experiences online.
- Is far higher than if I would have exchanged experiences online.

I think that the quality of my final product

- Is far lower than if I would have attended all lessons on location.
- Is lower than if I would have attended all lessons on location.
- Is equal to what I would have attended all lessons on location.
- Is higher than if I would have attended all lessons on location.
- Is far higher than if I would have attended all lessons on location.

I think that the insights gained during the course

- Can be applied far less in practice than if I would have attended all lessons on location.
- Can be applied less in practice than if I would have attended all lessons on location.
- Can be applied equally in practice than if I would have attended all lessons on location.
- Can be applied better in practice than if I would have attended all lessons on location.
- Can be applied far better in practice than if I would have attended all lessons on location.

I believe that, as a result of how the course was offered, balancing my educational program with my work and private life

- Was far less flexible than if all courses were on location.
- Was less flexible than if all courses were on location.
- Was equally flexible as if all courses were on location.
- Was more flexible than if all courses were on location.
- Was far more flexible than if all courses were on location.

Compared to other courses in the same period, my experiences with this specific course were:

- Far less
- Less
- Equal
- Better
- Far better
